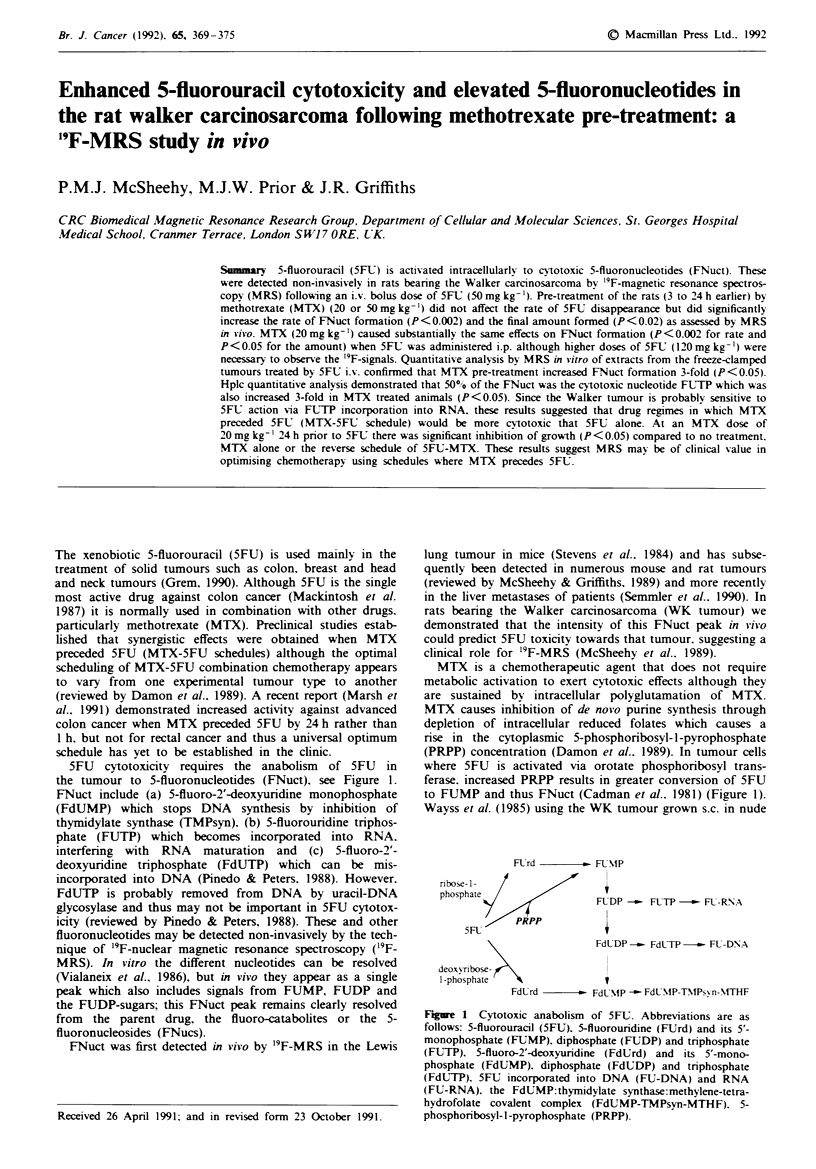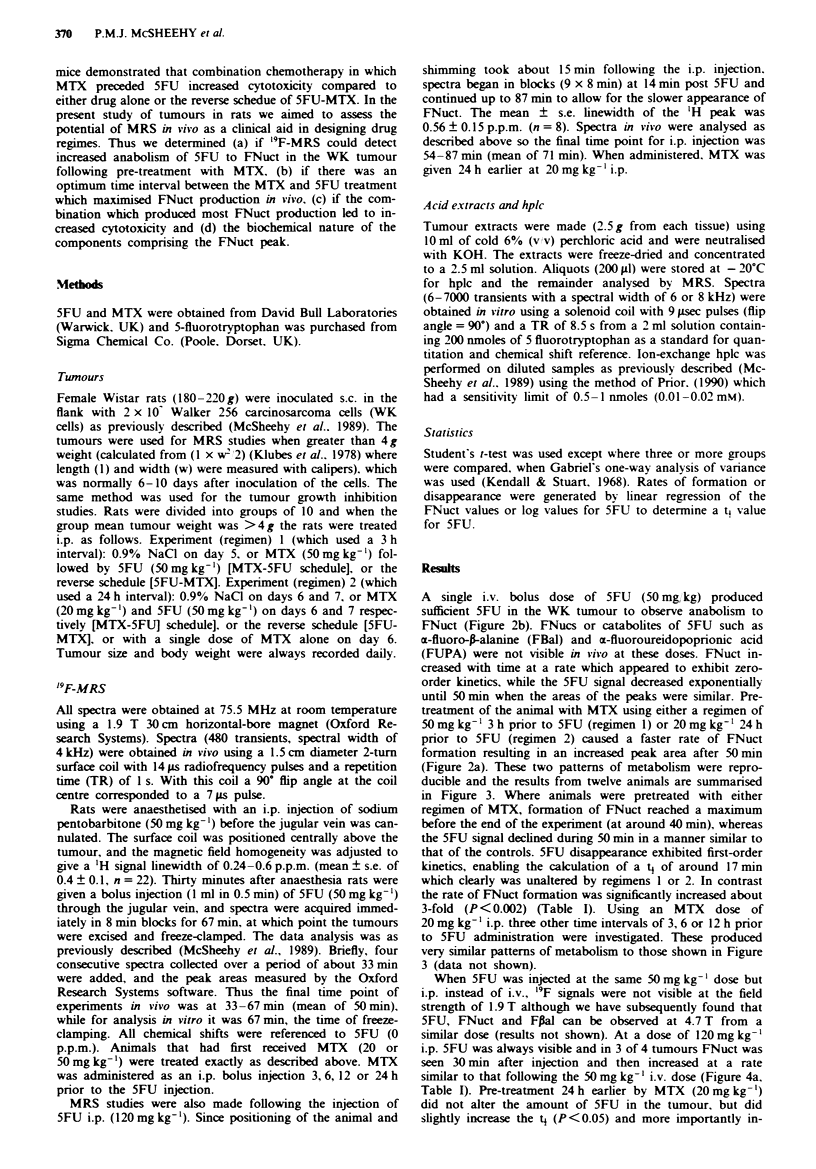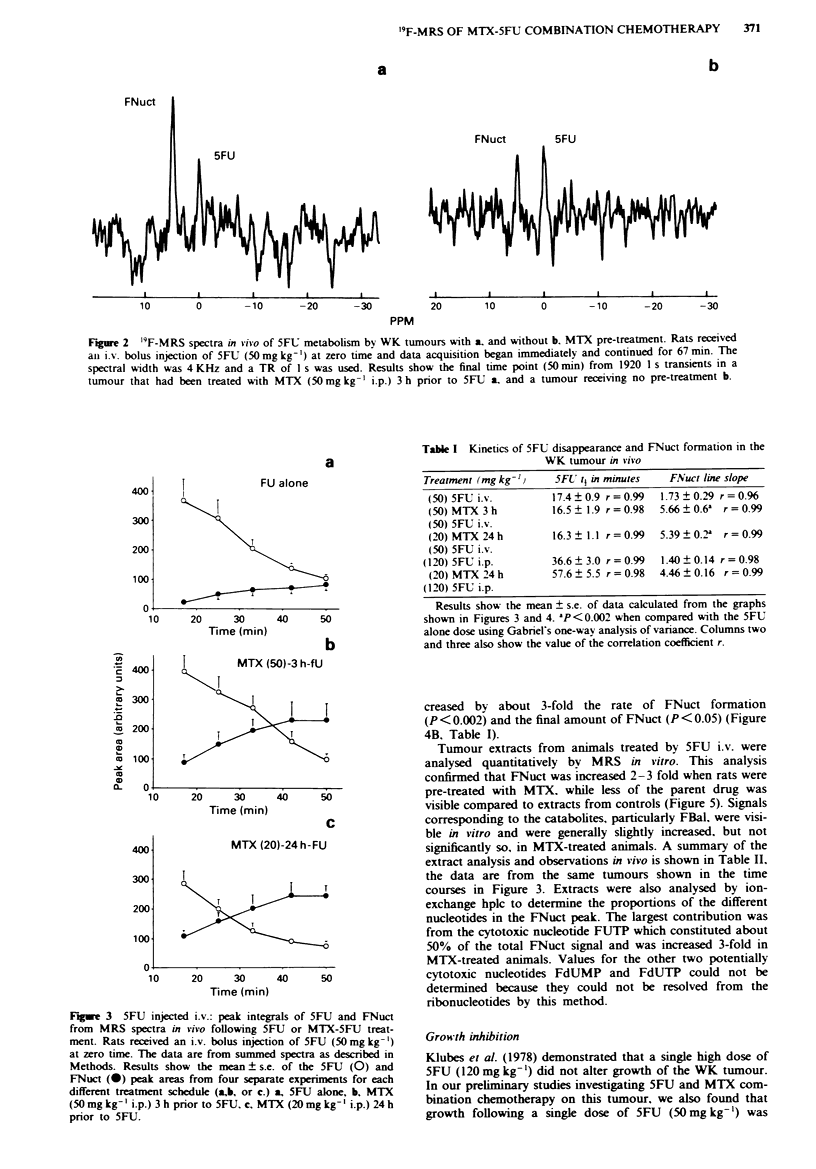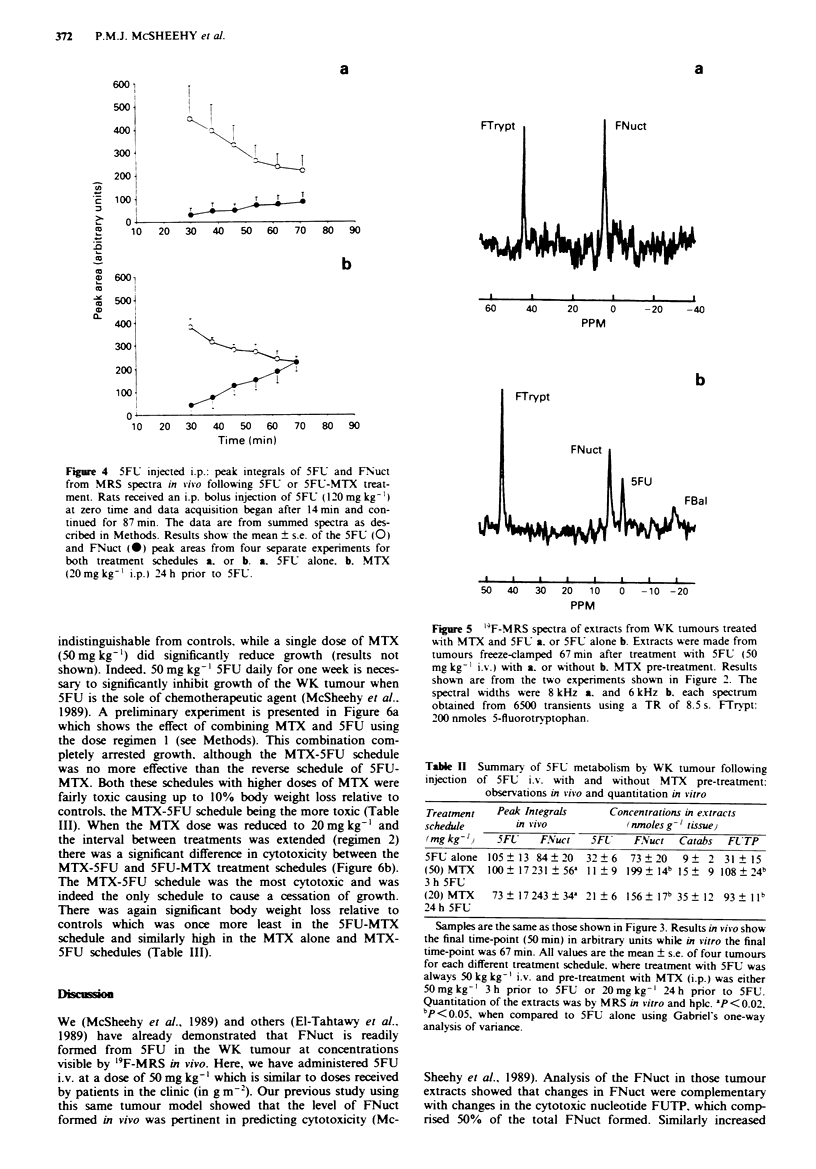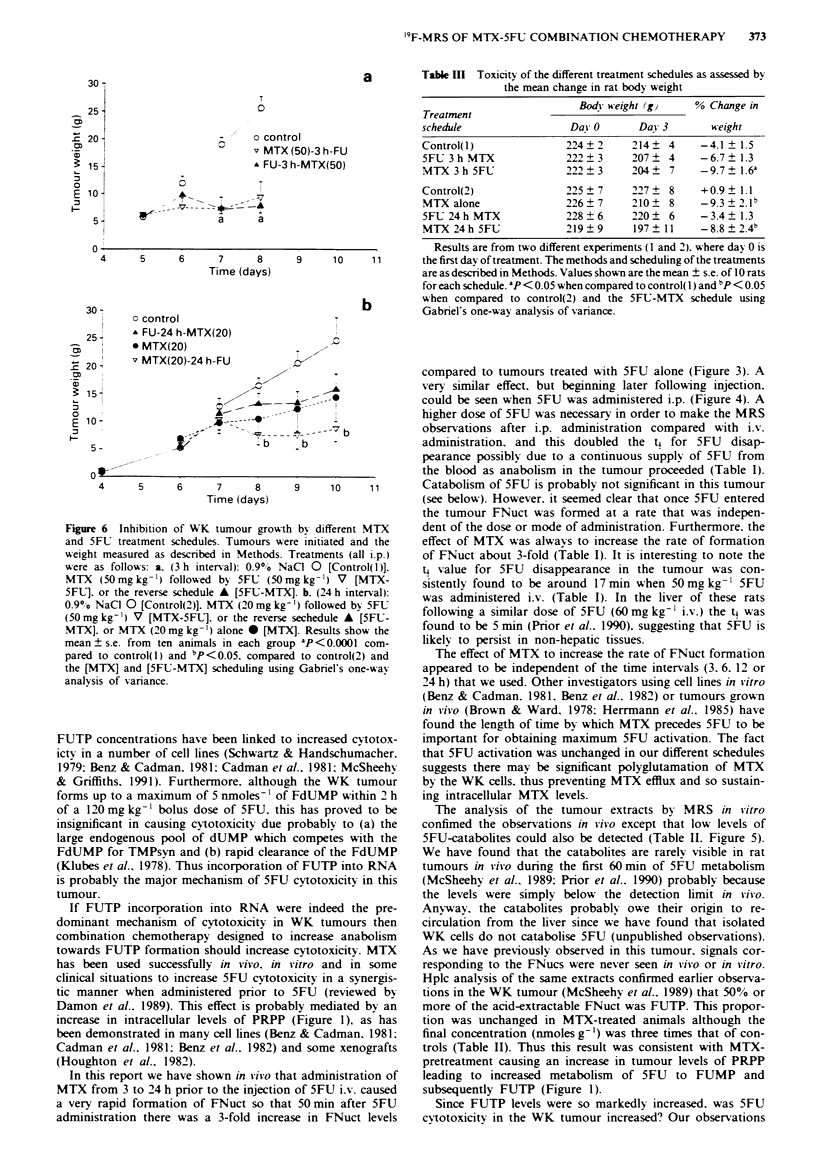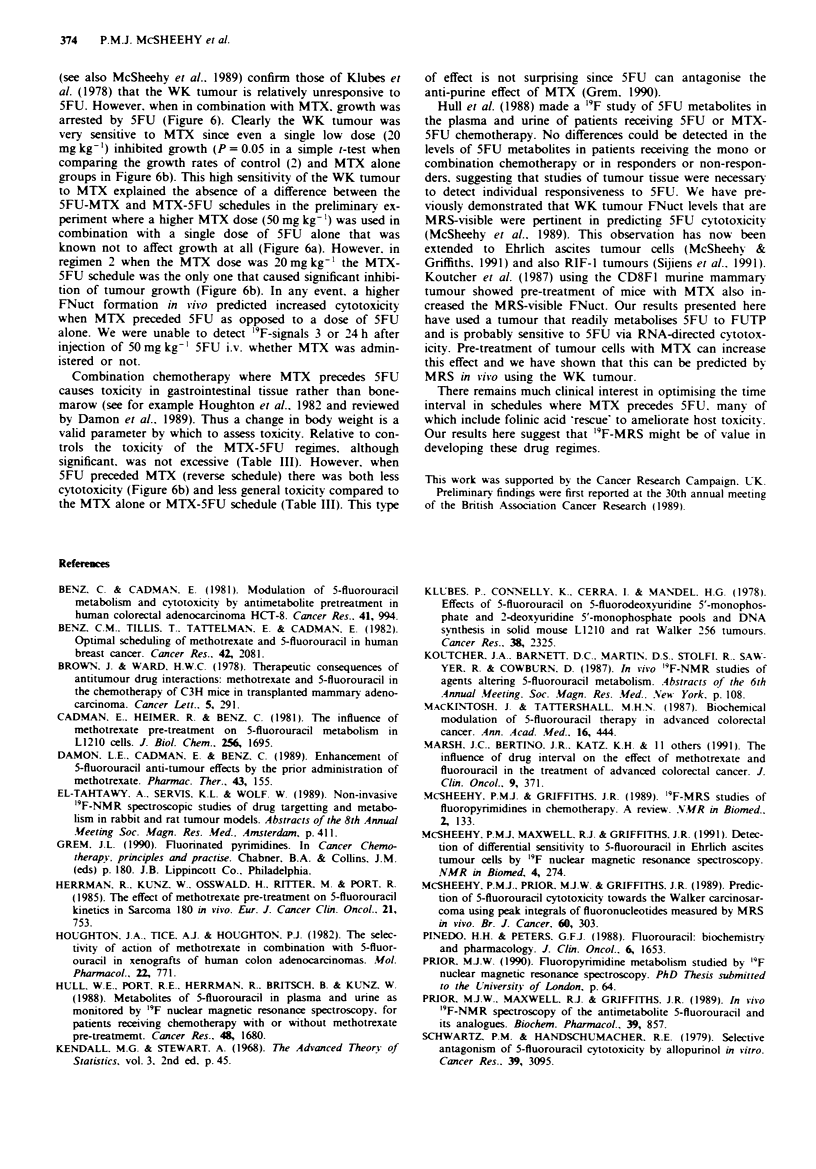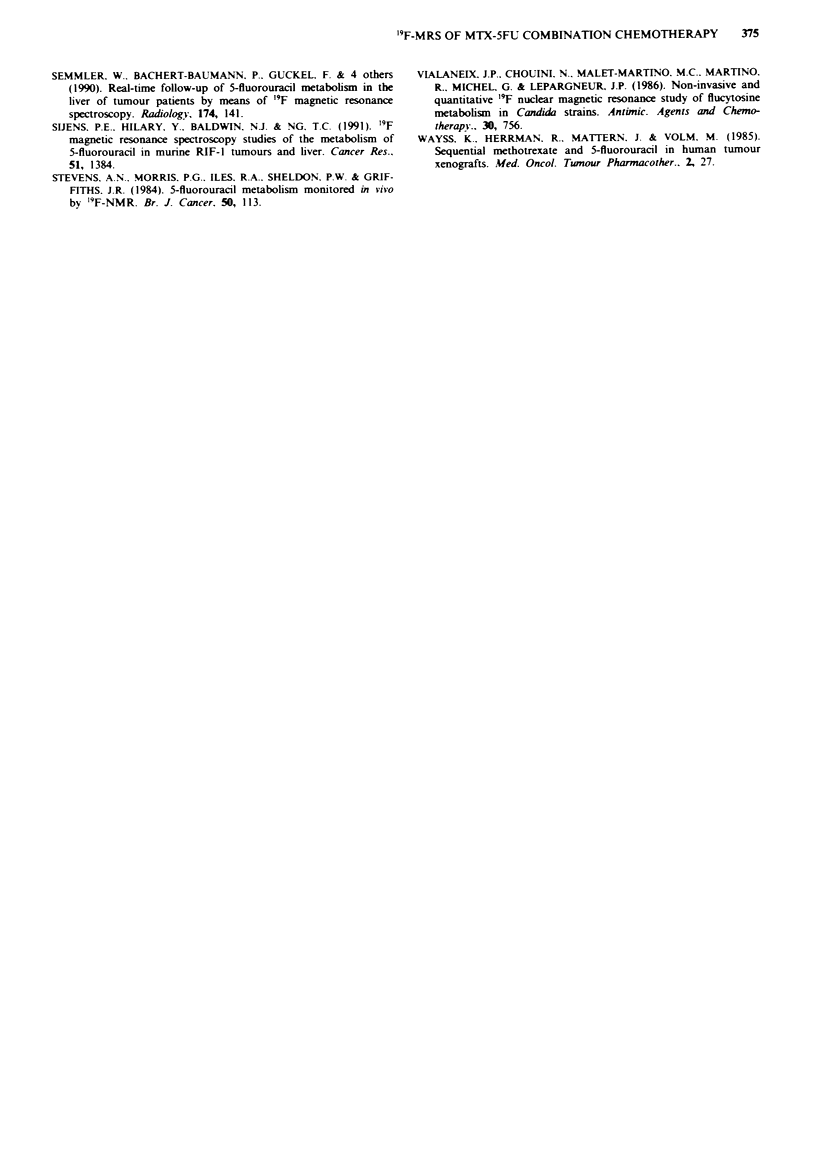# Enhanced 5-fluorouracil cytotoxicity and elevated 5-fluoronucleotides in the rat Walker carcinosarcoma following methotrexate pre-treatment: a 19F-MRS study in vivo.

**DOI:** 10.1038/bjc.1992.75

**Published:** 1992-03

**Authors:** P. M. McSheehy, M. J. Prior, J. R. Griffiths

**Affiliations:** Department of Cellular and Molecular Sciences, St. Georges Hospital Medical School, London, UK.

## Abstract

5-fluorouracil (5FU) is activated intracellularly to cytotoxic 5-fluoronucleotides (FNuct). These were detected non-invasively in rats bearing the Walker carcinosarcoma by 19F-magnetic resonance spectroscopy (MRS) following an i.v. bolus dose of 5FU (50 mg kg-1). Pre-treatment of the rats (3 to 24 h earlier) by methotrexate (MTX) (20 or 50 mg kg-1) did not affect the rate of 5FU disappearance but did significantly increase the rate of FNuct formation (P less than 0.002) and the final amount formed (P less than 0.02) as assessed by MRS in vivo. MTX (20 mg kg-1) caused substantially the same effects on FNuct formation (P less than 0.002 for rate and P less than 0.05 for the amount) when 5FU was administered i.p. although higher doses of 5FU (120 mg kg-1) were necessary to observe the 19F-signals. Quantitative analysis by MRS in vitro of extracts from the freeze-clamped tumours treated by 5FU i.v. confirmed that MTX pre-treatment increased FNuct formation 3-fold (P less than 0.05). Hplc quantitative analysis demonstrated that 50% of the FNuct was the cytotoxic nucleotide FUTP which was also increased 3-fold in MTX treated animals (P less than 0.05). Since the Walker tumour is probably sensitive to 5FU action via FUTP incorporation into RNA, these results suggested that drug regimes in which MTX preceded 5FU (MTX-5FU schedule) would be more cytotoxic that 5FU alone. At an MTX dose of 20 mg kg-1 24 h prior to 5FU there was significant inhibition of growth (P less than 0.05) compared to no treatment, MTX alone or the reverse schedule of 5FU-MTX. These results suggest MRS may be of clinical value in optimising chemotherapy using schedules where MTX precedes 5FU.


### Enhanced 5-fluorouracil cytotoxicity and elevated 5-fluoronucleotides in the rat walker carcinosarcoma following methotrexate pre-treatment: a $^{19}$F-MRS study *in vivo*

P.M.J. McSheehy, M.J.W. Prior & J.R. Griffiths

*CRC Biomedical Magnetic Resonance Research Group, Department of Cellular and Molecular Sciences, St. Georges Hospital Medical School, Cranmer Terrace, London SW17 0RE, UK.*

**Summary** 5-fluorouracil (5FU) is activated intracellularly to cytotoxic 5-fluoronucleotides (FNuct). These were detected non-invasively in rats bearing the Walker carcinosarcoma by $^{19}$F-magnetic resonance spectroscopy (MRS) following an i.v. bolus dose of 5FU (50 mg kg$^{-1}$). Pre-treatment of the rats (3 to 24 h earlier) by methotrexate (MTX) (20 or 50 mg kg$^{-1}$) did not affect the rate of 5FU disappearance but did significantly increase the rate of FNuct formation ($P<0.002$) and the final amount formed ($P<0.02$) as assessed by MRS *in vivo*. MTX (20 mg kg$^{-1}$) caused substantially the same effects on FNuct formation ($P<0.002$ for rate and $P<0.05$ for the amount) when 5FU was administered i.p. although higher doses of 5FU (120 mg kg$^{-1}$) were necessary to observe the $^{19}$F-signals. Quantitative analysis by MRS *in vitro* of extracts from the freeze-clamped tumours treated by 5FU i.v. confirmed that MTX pre-treatment increased FNuct formation 3-fold ($P<0.05$). Hplc quantitative analysis demonstrated that 50% of the FNuct was the cytotoxic nucleotide FUTP which was also increased 3-fold in MTX treated animals ($P<0.05$). Since the Walker tumour is probably sensitive to 5FU action via FUTP incorporation into RNA, these results suggested that drug regimes in which MTX preceded 5FU (MTX-5FU schedule) would be more cytotoxic that 5FU alone. At an MTX dose of 20 mg kg$^{-1}$ 24 h prior to 5FU there was significant inhibition of growth ($P<0.05$) compared to no treatment, MTX alone or the reverse schedule of 5FU-MTX. These results suggest MRS may be of clinical value in optimising chemotherapy using schedules where MTX precedes 5FU.

The xenobiotic 5-fluorouracil (5FU) is used mainly in the treatment of solid tumours such as colon, breast and head and neck tumours (Grem, 1990). Although 5FU is the single most active drug against colon cancer (Mackintosh *et al.* 1987) it is normally used in combination with other drugs, particularly methotrexate (MTX). Preclinical studies established that synergistic effects were obtained when MTX preceded 5FU (MTX-5FU schedules) although the optimal scheduling of MTX-5FU combination chemotherapy appears to vary from one experimental tumour type to another (reviewed by Damon *et al.*, 1989). A recent report (Marsh *et al.*, 1991) demonstrated increased activity against advanced colon cancer when MTX preceded 5FU by 24 h rather than 1 h, but not for rectal cancer and thus a universal optimum schedule has yet to be established in the clinic.

5FU cytotoxicity requires the anabolism of 5FU in the tumour to 5-fluoronucleotides (FNuct), see Figure 1. FNuct include (a) 5-fluoro-2′-deoxyuridine monophosphate (FdUMP) which stops DNA synthesis by inhibition of thymidylate synthase (TMPsyn), (b) 5-fluorouridine triphosphate (FUTP) which becomes incorporated into RNA, interfering with RNA maturation and (c) 5-fluoro-2′-deoxyuridine triphosphate (FdUTP) which can be misincorporated into DNA (Pinedo & Peters, 1988). However, FdUTP is probably removed from DNA by uracil-DNA glycosylase and thus may not be important in 5FU cytotoxicity (reviewed by Pinedo & Peters, 1988). These and other fluoronucleotides may be detected non-invasively by the technique of $^{19}$F-nuclear magnetic resonance spectroscopy ($^{19}$F-MRS). *In vitro* the different nucleotides can be resolved (Vialaneix *et al.*, 1986), but *in vivo* they appear as a single peak which also includes signals from FUMP, FUDP and the FUDP-sugars; this FNuct peak remains clearly resolved from the parent drug, the fluoro-catabolites or the 5-fluoronucleosides (FNucs).

FNuct was first detected *in vivo* by $^{19}$F-MRS in the Lewis lung tumour in mice (Stevens *et al.*, 1984) and has subsequently been detected in numerous mouse and rat tumours (reviewed by McSheehy & Griffiths, 1989) and more recently in the liver metastases of patients (Semmler *et al.*, 1990). In rats bearing the Walker carcinosarcoma (WK tumour) we demonstrated that the intensity of this FNuct peak *in vivo* could predict 5FU toxicity towards that tumour, suggesting a clinical role for $^{19}$F-MRS (McSheehy *et al.*, 1989).

MTX is a chemotherapeutic agent that does not require metabolic activation to exert cytotoxic effects although they are sustained by intracellular polyglutamation of MTX. MTX causes inhibition of *de novo* purine synthesis through depletion of intracellular reduced folates which causes a rise in the cytoplasmic 5-phosphoribosyl-1-pyrophosphate (PRPP) concentration (Damon *et al.*, 1989). In tumour cells where 5FU is activated via orotate phosphoribosyl transferase, increased PRPP results in greater conversion of 5FU to FUMP and thus FNuct (Cadman *et al.*, 1981) (Figure 1). Wayss *et al.* (1985) using the WK tumour grown s.c. in nude

**Figure 1** Cytotoxic anabolism of 5FU. Abbreviations are as follows: 5-fluorouracil (5FU), 5-fluorouridine (FUrd) and its 5′-monophosphate (FUMP), diphosphate (FUDP) and triphosphate (FUTP), 5-fluoro-2′-deoxyuridine (FdUrd) and its 5′-monophosphate (FdUMP), diphosphate (FdUDP) and triphosphate (FdUTP), 5FU incorporated into DNA (FU-DNA) and RNA (FU-RNA), the FdUMP:thymidylate synthase:methylene-tetrahydrofolate covalent complex (FdUMP-TMPsyn-MTHF), 5-phosphoribosyl-1-pyrophosphate (PRPP).

Received 26 April 1991; and in revised form 23 October 1991.

mice demonstrated that combination chemotherapy in which MTX preceded 5FU increased cytotoxicity compared to either drug alone or the reverse schedue of 5FU-MTX. In the present study of tumours in rats we aimed to assess the potential of MRS *in vivo* as a clinical aid in designing drug regimes. Thus we determined (a) if $^{19}$F-MRS could detect increased anabolism of 5FU to FNuct in the WK tumour following pre-treatment with MTX, (b) if there was an optimum time interval between the MTX and 5FU treatment which maximised FNuct production *in vivo*, (c) if the combination which produced most FNuct production led to increased cytotoxicity and (d) the biochemical nature of the components comprising the FNuct peak.

### Methods

5FU and MTX were obtained from David Bull Laboratories (Warwick, UK) and 5-fluorotryptophan was purchased from Sigma Chemical Co. (Poole, Dorset, UK).

### *Tumours*

Female Wistar rats (180–220 *g*) were inoculated s.c. in the flank with $2 \times 10^7$ Walker 256 carcinosarcoma cells (WK cells) as previously described (McSheehy *et al.*, 1989). The tumours were used for MRS studies when greater than 4 *g* weight (calculated from $(1 \times w^2/2)$ (Klubes *et al.*, 1978) where length (1) and width (w) were measured with calipers), which was normally 6–10 days after inoculation of the cells. The same method was used for the tumour growth inhibition studies. Rats were divided into groups of 10 and when the group mean tumour weight was $>4$ *g* the rats were treated i.p. as follows. Experiment (regimen) 1 (which used a 3 h interval): 0.9% NaCl on day 5, or MTX (50 mg kg$^{-1}$) followed by 5FU (50 mg kg$^{-1}$) [MTX-5FU schedule], or the reverse schedule [5FU-MTX]. Experiment (regimen) 2 (which used a 24 h interval): 0.9% NaCl on days 6 and 7, or MTX (20 mg kg$^{-1}$) and 5FU (50 mg kg$^{-1}$) on days 6 and 7 respectively [MTX-5FU] schedule], or the reverse schedule [5FU-MTX], or with a single dose of MTX alone on day 6. Tumour size and body weight were always recorded daily.

### *$^{19}$F-MRS*

All spectra were obtained at 75.5 MHz at room temperature using a 1.9 T 30 cm horizontal-bore magnet (Oxford Research Systems). Spectra (480 transients, spectral width of 4 kHz) were obtained *in vivo* using a 1.5 cm diameter 2-turn surface coil with 14 μs radiofrequency pulses and a repetition time (TR) of 1 s. With this coil a 90° flip angle at the coil centre corresponded to a 7 μs pulse.

Rats were anaesthetised with an i.p. injection of sodium pentobarbitone (50 mg kg$^{-1}$) before the jugular vein was cannulated. The surface coil was positioned centrally above the tumour, and the magnetic field homogeneity was adjusted to give a $^1$H signal linewidth of 0.24–0.6 p.p.m. (mean ± s.e. of 0.4 ± 0.1, $n = 22$). Thirty minutes after anaesthesia rats were given a bolus injection (1 ml in 0.5 min) of 5FU (50 mg kg$^{-1}$) through the jugular vein, and spectra were acquired immediately in 8 min blocks for 67 min, at which point the tumours were excised and freeze-clamped. The data analysis was as previously described (McSheehy *et al.*, 1989). Briefly, four consecutive spectra collected over a period of about 33 min were added, and the peak areas measured by the Oxford Research Systems software. Thus the final time point of experiments *in vivo* was at 33–67 min (mean of 50 min), while for analysis *in vitro* it was 67 min, the time of freeze-clamping. All chemical shifts were referenced to 5FU (0 p.p.m.). Animals that had first received MTX (20 or 50 mg kg$^{-1}$) were treated exactly as described above. MTX was administered as an i.p. bolus injection 3, 6, 12 or 24 h prior to the 5FU injection.

MRS studies were also made following the injection of 5FU i.p. (120 mg kg$^{-1}$). Since positioning of the animal and shimming took about 15 min following the i.p. injection, spectra began in blocks ($9 \times 8$ min) at 14 min post 5FU and continued up to 87 min to allow for the slower appearance of FNuct. The mean ± s.e. linewidth of the $^1$H peak was 0.56 ± 0.15 p.p.m. ($n = 8$). Spectra *in vivo* were analysed as described above so the final time point for i.p. injection was 54–87 min (mean of 71 min). When administered, MTX was given 24 h earlier at 20 mg kg$^{-1}$ i.p.

### *Acid extracts and hplc*

Tumour extracts were made (2.5 *g* from each tissue) using 10 ml of cold 6% (v/v) perchloric acid and were neutralised with KOH. The extracts were freeze-dried and concentrated to a 2.5 ml solution. Aliquots (200 μl) were stored at −20°C for hplc and the remainder analysed by MRS. Spectra (6–7000 transients with a spectral width of 6 or 8 kHz) were obtained *in vitro* using a solenoid coil with 9 μsec pulses (flip angle = 90°) and a TR of 8.5 s from a 2 ml solution containing 200 nmoles of 5 fluorotryptophan as a standard for quantitation and chemical shift reference. Ion-exchange hplc was performed on diluted samples as previously described (McSheehy *et al.*, 1989) using the method of Prior, (1990) which had a sensitivity limit of 0.5–1 nmoles (0.01–0.02 mM).

### *Statistics*

Student's *t*-test was used except where three or more groups were compared, when Gabriel's one-way analysis of variance was used (Kendall & Stuart, 1968). Rates of formation or disappearance were generated by linear regression of the FNuct values or log values for 5FU to determine a $t_{½}$ value for 5FU.

### Results

A single i.v. bolus dose of 5FU (50 mg/kg) produced sufficient 5FU in the WK tumour to observe anabolism to FNuct (Figure 2b). FNucs or catabolites of 5FU such as α-fluoro-β-alanine (FBal) and α-fluoroureidopoprionic acid (FUPA) were not visible *in vivo* at these doses. FNuct increased with time at a rate which appeared to exhibit zero-order kinetics, while the 5FU signal decreased exponentially until 50 min when the areas of the peaks were similar. Pretreatment of the animal with MTX using either a regimen of 50 mg kg$^{-1}$ 3 h prior to 5FU (regimen 1) or 20 mg kg$^{-1}$ 24 h prior to 5FU (regimen 2) caused a faster rate of FNuct formation resulting in an increased peak area after 50 min (Figure 2a). These two patterns of metabolism were reproducible and the results from twelve animals are summarised in Figure 3. Where animals were pretreated with either regimen of MTX, formation of FNuct reached a maximum before the end of the experiment (at around 40 min), whereas the 5FU signal declined during 50 min in a manner similar to that of the controls. 5FU disappearance exhibited first-order kinetics, enabling the calculation of a $t_{½}$ of around 17 min which clearly was unaltered by regimens 1 or 2. In contrast the rate of FNuct formation was significantly increased about 3-fold ($P<0.002$) (Table I). Using an MTX dose of 20 mg kg$^{-1}$ i.p. three other time intervals of 3, 6 or 12 h prior to 5FU administration were investigated. These produced very similar patterns of metabolism to those shown in Figure 3 (data not shown).

When 5FU was injected at the same 50 mg kg$^{-1}$ dose but i.p. instead of i.v., $^{19}$F signals were not visible at the field strength of 1.9 T although we have subsequently found that 5FU, FNuct and FβaI can be observed at 4.7 T from a similar dose (results not shown). At a dose of 120 mg kg$^{-1}$ i.p. 5FU was always visible and in 3 of 4 tumours FNuct was seen 30 min after injection and then increased at a rate similar to that following the 50 mg kg$^{-1}$ i.v. dose (Figure 4a, Table I). Pre-treatment 24 h earlier by MTX (20 mg kg$^{-1}$) did not alter the amount of 5FU in the tumour, but did slightly increase the $t_{½}$ ($P<0.05$) and more importantly in-

**Figure 2** $^{19}$F-MRS spectra *in vivo* of 5FU metabolism by WK tumours with **a**, and without **b**, MTX pre-treatment. Rats received an i.v. bolus injection of 5FU (50 mg kg$^{-1}$) at zero time and data acquisition began immediately and continued for 67 min. The spectral width was 4 KHz and a TR of 1 s was used. Results show the final time point (50 min) from 1920 1 s transients in a tumour that had been treated with MTX (50 mg kg$^{-1}$ i.p.) 3 h prior to 5FU **a**, and a tumour receiving no pre-treatment **b**.

**Figure 3** 5FU injected i.v.: peak integrals of 5FU and FNuct from MRS spectra *in vivo* following 5FU or MTX-5FU treatment. Rats received an i.v. bolus injection of 5FU (50 mg kg$^{-1}$) at zero time. The data are from summed spectra as described in Methods. Results show the mean ± s.e. of the 5FU (O) and FNuct (●) peak areas from four separate experiments for each different treatment schedule (**a**,**b**, or **c**.) **a**, 5FU alone, **b**, MTX (50 mg kg$^{-1}$ i.p.) 3 h prior to 5FU, **c**, MTX (20 mg kg$^{-1}$ i.p.) 24 h prior to 5FU.

**Table I** Kinetics of 5FU disappearance and FNuct formation in the WK tumour *in vivo*

| *Treatment (mg kg$^{-1}$)* | *5FU $t_{\frac{1}{2}}$ in minutes* | *FNuct line slope* |
|---|---|---|
| (50) 5FU i.v. | 17.4 ± 0.9 $r = 0.99$ | 1.73 ± 0.29 $r = 0.96$ |
| (50) MTX 3 h<br>(50) 5FU i.v. | 16.5 ± 1.9 $r = 0.98$ | 5.66 ± 0.6$^{a}$ $r = 0.99$ |
| (20) MTX 24 h<br>(50) 5FU i.v. | 16.3 ± 1.1 $r = 0.99$ | 5.39 ± 0.2$^{a}$ $r = 0.99$ |
| (120) 5FU i.p. | 36.6 ± 3.0 $r = 0.99$ | 1.40 ± 0.14 $r = 0.98$ |
| (20) MTX 24 h<br>(120) 5FU i.p. | 57.6 ± 5.5 $r = 0.98$ | 4.46 ± 0.16 $r = 0.99$ |

Results show the mean ± s.e. of data calculated from the graphs shown in Figures 3 and 4. $^{a}P < 0.002$ when compared with the 5FU alone dose using Gabriel's one-way analysis of variance. Columns two and three also show the value of the correlation coefficient $r$.

creased by about 3-fold the rate of FNuct formation ($P < 0.002$) and the final amount of FNuct ($P < 0.05$) (Figure 4B, Table I).

Tumour extracts from animals treated by 5FU i.v. were analysed quantitatively by MRS *in vitro*. This analysis confirmed that FNuct was increased 2–3 fold when rats were pre-treated with MTX, while less of the parent drug was visible compared to extracts from controls (Figure 5). Signals corresponding to the catabolites, particularly FBal, were visible *in vitro* and were generally slightly increased, but not significantly so, in MTX-treated animals. A summary of the extract analysis and observations *in vivo* is shown in Table II, the data are from the same tumours shown in the time courses in Figure 3. Extracts were also analysed by ion-exchange hplc to determine the proportions of the different nucleotides in the FNuct peak. The largest contribution was from the cytotoxic nucleotide FUTP which constituted about 50% of the total FNuct signal and was increased 3-fold in MTX-treated animals. Values for the other two potentially cytotoxic nucleotides FdUMP and FdUTP could not be determined because they could not be resolved from the ribonucleotides by this method.

### *Growth inhibition*

Klubes *et al.* (1978) demonstrated that a single high dose of 5FU (120 mg kg$^{-1}$) did not alter growth of the WK tumour. In our preliminary studies investigating 5FU and MTX combination chemotherapy on this tumour, we also found that growth following a single dose of 5FU (50 mg kg$^{-1}$) was

**Figure 4** 5FU injected i.p.: peak integrals of 5FU and FNuct from MRS spectra *in vivo* following 5FU or 5FU-MTX treatment. Rats received an i.p. bolus injection of 5FU (120 mg kg$^{-1}$) at zero time and data acquisition began after 14 min and continued for 87 min. The data are from summed spectra as described in Methods. Results show the mean ± s.e. of the 5FU (○) and FNuct (●) peak areas from four separate experiments for both treatment schedules **a**, or **b**. **a**, 5FU alone. **b**, MTX (20 mg kg$^{-1}$ i.p.) 24 h prior to 5FU.

**Figure 5** $^{19}$F-MRS spectra of extracts from WK tumours treated with MTX and 5FU **a**, or 5FU alone **b**. Extracts were made from tumours freeze-clamped 67 min after treatment with 5FU (50 mg kg$^{-1}$ i.v.) with **a**, or without **b**, MTX pre-treatment. Results shown are from the two experiments shown in Figure 2. The spectral widths were 8 kHz **a**, and 6 kHz **b**, each spectrum obtained from 6500 transients using a TR of 8.5 s. FTrypt: 200 nmoles 5-fluorotryptophan.

indistinguishable from controls, while a single dose of MTX (50 mg kg$^{-1}$) did significantly reduce growth (results not shown). Indeed, 50 mg kg$^{-1}$ 5FU daily for one week is necessary to significantly inhibit growth of the WK tumour when 5FU is the sole of chemotherapeutic agent (McSheehy *et al.*, 1989). A preliminary experiment is presented in Figure 6a which shows the effect of combining MTX and 5FU using the dose regimen 1 (see Methods). This combination completely arrested growth, although the MTX-5FU schedule was no more effective than the reverse schedule of 5FU-MTX. Both these schedules with higher doses of MTX were fairly toxic causing up to 10% body weight loss relative to controls, the MTX-5FU schedule being the more toxic (Table III). When the MTX dose was reduced to 20 mg kg$^{-1}$ and the interval between treatments was extended (regimen 2) there was a significant difference in cytotoxicity between the MTX-5FU and 5FU-MTX treatment schedules (Figure 6b). The MTX-5FU schedule was the most cytotoxic and was indeed the only schedule to cause a cessation of growth. There was again significant body weight loss relative to controls which was once more least in the 5FU-MTX schedule and similarly high in the MTX alone and MTX-5FU schedules (Table III).

### Discussion

We (McSheehy *et al.*, 1989) and others (El-Tahtawy *et al.*, 1989) have already demonstrated that FNuct is readily formed from 5FU in the WK tumour at concentrations visible by $^{19}$F-MRS *in vivo*. Here, we have administered 5FU i.v. at a dose of 50 mg kg$^{-1}$ which is similar to doses received by patients in the clinic (in g m$^{-2}$). Our previous study using this same tumour model showed that the level of FNuct formed *in vivo* was pertinent in predicting cytotoxicity (McSheehy *et al.*, 1989). Analysis of the FNuct in those tumour extracts showed that changes in FNuct were complementary with changes in the cytotoxic nucleotide FUTP, which comprised 50% of the total FNuct formed. Similarly increased

**Table II** Summary of 5FU metabolism by WK tumour following injection of 5FU i.v. with and without MTX pre-treatment: observations *in vivo* and quantitation *in vitro*

| *Treatment schedule* | *Peak Integrals in vivo* | | *Concentrations in extracts (nmoles g$^{-1}$ tissue)* | | | |
|---|---|---|---|---|---|---|
| *(mg kg$^{-1}$)* | *5FU* | *FNuct* | *5FU* | *FNuct* | *Catabs* | *FUTP* |
| 5FU alone | 105 ± 13 | 84 ± 20 | 32 ± 6 | 73 ± 20 | 9 ± 2 | 31 ± 15 |
| (50) MTX 3 h 5FU | 100 ± 17 | 231 ± 56[a] | 11 ± 9 | 199 ± 14[b] | 15 ± 9 | 108 ± 24[b] |
| (20) MTX 24 h 5FU | 73 ± 17 | 243 ± 34[a] | 21 ± 6 | 156 ± 17[b] | 35 ± 12 | 93 ± 11[b] |

Samples are the same as those shown in Figure 3. Results *in vivo* show the final time-point (50 min) in arbitrary units while *in vitro* the final time-point was 67 min. All values are the mean ± s.e. of four tumours for each different treatment schedule, where treatment with 5FU was always 50 kg kg$^{-1}$ i.v. and pre-treatment with MTX (i.p.) was either 50 mg kg$^{-1}$ 3 h prior to 5FU or 20 mg kg$^{-1}$ 24 h prior to 5FU. Quantitation of the extracts was by MRS *in vitro* and hplc. [a]$P < 0.02$, [b]$P < 0.05$, when compared to 5FU alone using Gabriel's one-way analysis of variance.

**Figure 6** Inhibition of WK tumour growth by different MTX and 5FU treatment schedules. Tumours were initiated and the weight measured as described in Methods. Treatments (all i.p.) were as follows: **a**, (3 h interval): 0.9% NaCl ○ [Control(1)], MTX (50 mg $kg^{-1}$) followed by 5FU (50 mg $kg^{-1}$) ▽ [MTX-5FU], or the reverse schedule ▲ [5FU-MTX]. **b**, (24 h interval): 0.9% NaCl ○ [Control(2)], MTX (20 mg $kg^{-1}$) followed by 5FU (50 mg $kg^{-1}$) ▽ [MTX-5FU], or the reverse sechedule ▲ [5FU-MTX], or MTX (20 mg $kg^{-1}$) alone ● [MTX]. Results show the mean ± s.e. from ten animals in each group $^{a}P<0.0001$ compared to control(1) and $^{b}P<0.05$, compared to control(2) and the [MTX] and [5FU-MTX] scheduling using Gabriel's one-way analysis of variance.

**Table III** Toxicity of the different treatment schedules as assessed by the mean change in rat body weight

| Treatment schedule | Body weight (g) Day 0 | Body weight (g) Day 3 | % Change in weight |
|---|---|---|---|
| Control(1) | 224 ± 2 | 214 ± 4 | −4.1 ± 1.5 |
| 5FU 3 h MTX | 222 ± 3 | 207 ± 4 | −6.7 ± 1.3 |
| MTX 3 h 5FU | 222 ± 3 | 204 ± 7 | $-9.7 \pm 1.6^{a}$ |
| Control(2) | 225 ± 7 | 227 ± 8 | +0.9 ± 1.1 |
| MTX alone | 226 ± 7 | 210 ± 8 | $-9.3 \pm 2.1^{b}$ |
| 5FU 24 h MTX | 228 ± 6 | 220 ± 6 | −3.4 ± 1.3 |
| MTX 24 h 5FU | 219 ± 9 | 197 ± 11 | $-8.8 \pm 2.4^{b}$ |

Results are from two different experiments (1 and 2), where day 0 is the first day of treatment. The methods and scheduling of the treatments are as described in Methods. Values shown are the mean ± s.e. of 10 rats for each schedule. $^{a}P<0.05$ when compared to control(1) and $^{b}P<0.05$ when compared to control(2) and the 5FU-MTX schedule using Gabriel's one-way analysis of variance.

FUTP concentrations have been linked to increased cytotoxicty in a number of cell lines (Schwartz & Handschumacher, 1979; Benz & Cadman, 1981; Cadman *et al.*, 1981; McSheehy & Griffiths, 1991). Furthermore, although the WK tumour forms up to a maximum of 5 $nmoles^{-1}$ of FdUMP within 2 h of a 120 mg $kg^{-1}$ bolus dose of 5FU, this has proved to be insignificant in causing cytotoxicity due probably to (a) the large endogenous pool of dUMP which competes with the FdUMP for TMPsyn and (b) rapid clearance of the FdUMP (Klubes *et al.*, 1978). Thus incorporation of FUTP into RNA is probably the major mechanism of 5FU cytotoxicity in this tumour.

If FUTP incorporation into RNA were indeed the predominant mechanism of cytotoxicity in WK tumours then combination chemotherapy designed to increase anabolism towards FUTP formation should increase cytotoxicity. MTX has been used successfully *in vivo*, *in vitro* and in some clinical situations to increase 5FU cytotoxicity in a synergistic manner when administered prior to 5FU (reviewed by Damon *et al.*, 1989). This effect is probably mediated by an increase in intracellular levels of PRPP (Figure 1), as has been demonstrated in many cell lines (Benz & Cadman, 1981; Cadman *et al.*, 1981; Benz *et al.*, 1982) and some xenografts (Houghton *et al.*, 1982).

In this report we have shown *in vivo* that administration of MTX from 3 to 24 h prior to the injection of 5FU i.v. caused a very rapid formation of FNuct so that 50 min after 5FU administration there was a 3-fold increase in FNuct levels compared to tumours treated with 5FU alone (Figure 3). A very similar effect, but beginning later following injection, could be seen when 5FU was administered i.p. (Figure 4). A higher dose of 5FU was necessary in order to make the MRS observations after i.p. administration compared with i.v. administration, and this doubled the $t_{\frac{1}{2}}$ for 5FU disappearance possibly due to a continuous supply of 5FU from the blood as anabolism in the tumour proceeded (Table I). Catabolism of 5FU is probably not significant in this tumour (see below). However, it seemed clear that once 5FU entered the tumour FNuct was formed at a rate that was independent of the dose or mode of administration. Furthermore, the effect of MTX was always to increase the rate of formation of FNuct about 3-fold (Table I). It is interesting to note the $t_{\frac{1}{2}}$ value for 5FU disappearance in the tumour was consistently found to be around 17 min when 50 mg $kg^{-1}$ 5FU was administered i.v. (Table I). In the liver of these rats following a similar dose of 5FU (60 mg $kg^{-1}$ i.v.) the $t_{\frac{1}{2}}$ was found to be 5 min (Prior *et al.*, 1990), suggesting that 5FU is likely to persist in non-hepatic tissues.

The effect of MTX to increase the rate of FNuct formation appeared to be independent of the time intervals (3, 6, 12 or 24 h) that we used. Other investigators using cell lines *in vitro* (Benz & Cadman, 1981, Benz *et al.*, 1982) or tumours grown *in vivo* (Brown & Ward, 1978; Herrmann *et al.*, 1985) have found the length of time by which MTX precedes 5FU to be important for obtaining maximum 5FU activation. The fact that 5FU activation was unchanged in our different schedules suggests there may be significant polyglutamation of MTX by the WK cells, thus preventing MTX efflux and so sustaining intracellular MTX levels.

The analysis of the tumour extracts by MRS *in vitro* confimed the observations *in vivo* except that low levels of 5FU-catabolites could also be detected (Table II, Figure 5). We have found that the catabolites are rarely visible in rat tumours *in vivo* during the first 60 min of 5FU metabolism (McSheehy *et al.*, 1989; Prior *et al.*, 1990) probably because the levels were simply below the detection limit *in vivo*. Anyway, the catabolites probably owe their origin to recirculation from the liver since we have found that isolated WK cells do not catabolise 5FU (unpublished observations). As we have previously observed in this tumour, signals corresponding to the FNucs were never seen *in vivo* or *in vitro*. Hplc analysis of the same extracts confirmed earlier observations in the WK tumour (McSheehy *et al.*, 1989) that 50% or more of the acid-extractable FNuct was FUTP. This proportion was unchanged in MTX-treated animals although the final concentration (nmoles $g^{-1}$) was three times that of controls (Table II). Thus this result was consistent with MTX-pretreatment causing an increase in tumour levels of PRPP leading to increased metabolism of 5FU to FUMP and subsequently FUTP (Figure 1).

Since FUTP levels were so markedly increased, was 5FU cytotoxicity in the WK tumour increased? Our observations

(see also McSheehy *et al.*, 1989) confirm those of Klubes *et al.* (1978) that the WK tumour is relatively unresponsive to 5FU. However, when in combination with MTX, growth was arrested by 5FU (Figure 6). Clearly the WK tumour was very sensitive to MTX since even a single low dose (20 mg kg$^{-1}$) inhibited growth ($P = 0.05$ in a simple *t*-test when comparing the growth rates of control (2) and MTX alone groups in Figure 6b). This high sensitivity of the WK tumour to MTX explained the absence of a difference between the 5FU-MTX and MTX-5FU schedules in the preliminary experiment where a higher MTX dose (50 mg kg$^{-1}$) was used in combination with a single dose of 5FU alone that was known not to affect growth at all (Figure 6a). However, in regimen 2 when the MTX dose was 20 mg kg$^{-1}$ the MTX-5FU schedule was the only one that caused significant inhibition of tumour growth (Figure 6b). In any event, a higher FNuct formation *in vivo* predicted increased cytotoxicity when MTX preceded 5FU as opposed to a dose of 5FU alone. We were unable to detect $^{19}$F-signals 3 or 24 h after injection of 50 mg kg$^{-1}$ 5FU i.v. whether MTX was administered or not.

Combination chemotherapy where MTX precedes 5FU causes toxicity in gastrointestinal tissue rather than bone-marow (see for example Houghton *et al.*, 1982 and reviewed by Damon *et al.*, 1989). Thus a change in body weight is a valid parameter by which to assess toxicity. Relative to controls the toxicity of the MTX-5FU regimes, although significant, was not excessive (Table III). However, when 5FU preceded MTX (reverse schedule) there was both less cytotoxicity (Figure 6b) and less general toxicity compared to the MTX alone or MTX-5FU schedule (Table III). This type of effect is not surprising since 5FU can antagonise the anti-purine effect of MTX (Grem, 1990).

Hull *et al.* (1988) made a $^{19}$F study of 5FU metabolites in the plasma and urine of patients receiving 5FU or MTX-5FU chemotherapy. No differences could be detected in the levels of 5FU metabolites in patients receiving the mono or combination chemotherapy or in responders or non-responders, suggesting that studies of tumour tissue were necessary to detect individual responsiveness to 5FU. We have previously demonstrated that WK tumour FNuct levels that are MRS-visible were pertinent in predicting 5FU cytotoxicity (McSheehy *et al.*, 1989). This observation has now been extended to Ehrlich ascites tumour cells (McSheehy & Griffiths, 1991) and also RIF-1 tumours (Sijiens *et al.*, 1991). Koutcher *et al.* (1987) using the CD8F1 murine mammary tumour showed pre-treatment of mice with MTX also increased the MRS-visible FNuct. Our results presented here have used a tumour that readily metabolises 5FU to FUTP and is probably sensitive to 5FU via RNA-directed cytotoxicity. Pre-treatment of tumour cells with MTX can increase this effect and we have shown that this can be predicted by MRS *in vivo* using the WK tumour.

There remains much clinical interest in optimising the time interval in schedules where MTX precedes 5FU, many of which include folinic acid 'rescue' to ameliorate host toxicity. Our results here suggest that $^{19}$F-MRS might be of value in developing these drug regimes.

This work was supported by the Cancer Research Campaign, UK. Preliminary findings were first reported at the 30th annual meeting of the British Association Cancer Research (1989).

### References

BENZ, C. & CADMAN, E. (1981). Modulation of 5-fluorouracil metabolism and cytotoxicity by antimetabolite pretreatment in human colorectal adenocarcinoma HCT-8. *Cancer Res.*, **41**, 994.

BENZ, C.M., TILLIS, T., TATTELMAN, E. & CADMAN, E. (1982). Optimal scheduling of methotrexate and 5-fluorouracil in human breast cancer. *Cancer Res.*, **42**, 2081.

BROWN, J. & WARD, H.W.C. (1978). Therapeutic consequences of antitumour drug interactions: methotrexate and 5-fluorouracil in the chemotherapy of C3H mice in transplanted mammary adenocarcinoma. *Cancer Lett.*, **5**, 291.

CADMAN, E., HEIMER, R. & BENZ, C. (1981). The influence of methotrexate pre-treatment on 5-fluorouracil metabolism in L1210 cells. *J. Biol. Chem.*, **256**, 1695.

DAMON, L.E., CADMAN, E. & BENZ, C. (1989). Enhancement of 5-fluorouracil anti-tumour effects by the prior administration of methotrexate. *Pharmac. Ther.*, **43**, 155.

EL-TAHTAWY, A., SERVIS, K.L. & WOLF, W. (1989). Non-invasive $^{19}$F-NMR spectroscopic studies of drug targetting and metabolism in rabbit and rat tumour models. *Abstracts of the 8th Annual Meeting Soc. Magn. Res. Med., Amsterdam*, p. 411.

GREM, J.L. (1990). Fluorinated pyrimidines. In *Cancer Chemotherapy, principles and practise*. Chabner, B.A. & Collins, J.M. (eds) p. 180. J.B. Lippincott Co., Philadelphia.

HERRMAN, R., KUNZ, W., OSSWALD, H., RITTER, M. & PORT, R. (1985). The effect of methotrexate pre-treatment on 5-fluorouracil kinetics in Sarcoma 180 *in vivo*. *Eur. J. Cancer Clin. Oncol.*, **21**, 753.

HOUGHTON, J.A., TICE, A.J. & HOUGHTON, P.J. (1982). The selectivity of action of methotrexate in combination with 5-fluorouracil in xenografts of human colon adenocarcinomas. *Mol. Pharmacol.*, **22**, 771.

HULL, W.E., PORT, R.E., HERRMAN, R., BRITSCH, B. & KUNZ, W. (1988). Metabolites of 5-fluorouracil in plasma and urine as monitored by $^{19}$F nuclear magnetic resonance spectroscopy, for patients receiving chemotherapy with or without methotrexate pre-treatmemt. *Cancer Res.*, **48**, 1680.

KENDALL, M.G. & STEWART, A. (1968). *The Advanced Theory of Statistics*, vol. 3, 2nd ed, p. 45.

KLUBES, P., CONNELLY, K., CERRA, I. & MANDEL, H.G. (1978). Effects of 5-fluorouracil on 5-fluorodeoxyuridine 5'-monophosphate and 2-deoxyuridine 5'-monophosphate pools and DNA synthesis in solid mouse L1210 and rat Walker 256 tumours. *Cancer Res.*, **38**, 2325.

KOUTCHER, J.A., BARNETT, D.C., MARTIN, D.S., STOLFI, R., SAWYER, R. & COWBURN, D. (1987). *In vivo* $^{19}$F-NMR studies of agents altering 5-fluorouracil metabolism. *Abstracts of the 6th Annual Meeting, Soc. Magn. Res. Med., New York*, p. 108.

MACKINTOSH, J. & TATTERSHALL, M.H.N. (1987). Biochemical modulation of 5-fluorouracil therapy in advanced colorectal cancer. *Ann. Acad. Med.*, **16**, 444.

MARSH, J.C., BERTINO, J.R., KATZ, K.H. & 11 others (1991). The influence of drug interval on the effect of methotrexate and fluorouracil in the treatment of advanced colorectal cancer. *J. Clin. Oncol.*, **9**, 371.

MCSHEEHY, P.M.J. & GRIFFITHS, J.R. (1989). $^{19}$F-MRS studies of fluoropyrimidines in chemotherapy. A review. *NMR in Biomed.*, **2**, 133.

MCSHEEHY, P.M.J, MAXWELL, R.J. & GRIFFITHS, J.R. (1991). Detection of differential sensitivity to 5-fluorouracil in Ehrlich ascites tumour cells by $^{19}$F nuclear magnetic resonance spectroscopy. *NMR in Biomed*, **4**, 274.

MCSHEEHY, P.M.J., PRIOR, M.J.W. & GRIFFITHS, J.R. (1989). Prediction of 5-fluorouracil cytotoxicity towards the Walker carcinosarcoma using peak integrals of fluoronucleotides measured by MRS *in vivo*. *Br. J. Cancer*, **60**, 303.

PINEDO, H.H. & PETERS, G.F.J. (1988). Fluorouracil: biochemistry and pharmacology. *J. Clin. Oncol.*, **6**, 1653.

PRIOR, M.J.W. (1990). Fluoropyrimidine metabolism studied by $^{19}$F nuclear magnetic resonance spectroscopy. *PhD Thesis submitted to the University of London*, p. 64.

PRIOR, M.J.W., MAXWELL, R.J. & GRIFFITHS, J.R. (1989). *In vivo* $^{19}$F-NMR spectroscopy of the antimetabolite 5-fluorouracil and its analogues. *Biochem. Pharmacol.*, **39**, 857.

SCHWARTZ, P.M. & HANDSCHUMACHER, R.E. (1979). Selective antagonism of 5-fluorouracil cytotoxicity by allopurinol *in vitro*. *Cancer Res.*, **39**, 3095.

SEMMLER, W., BACHERT-BAUMANN, P., GUCKEL, F. & 4 others (1990). Real-time follow-up of 5-fluorouracil metabolism in the liver of tumour patients by means of $^{19}$F magnetic resonance spectroscopy. *Radiology*, **174**, 141.

SIJENS, P.E., HILARY, Y., BALDWIN, N.J. & NG, T.C. (1991). $^{19}$F magnetic resonance spectroscopy studies of the metabolism of 5-fluorouracil in murine RIF-1 tumours and liver. *Cancer Res.*, **51**, 1384.

STEVENS, A.N., MORRIS, P.G., ILES, R.A., SHELDON, P.W. & GRIFFITHS, J.R. (1984). 5-fluorouracil metabolism monitored *in vivo* by $^{19}$F-NMR. *Br. J. Cancer*, **50**, 113.

VIALANEIX, J.P., CHOUINI, N., MALET-MARTINO, M.C., MARTINO, R., MICHEL, G. & LEPARGNEUR, J.P. (1986). Non-invasive and quantitative $^{19}$F nuclear magnetic resonance study of flucytosine metabolism in *Candida* strains. *Antimic. Agents and Chemotherapy*., **30**, 756.

WAYSS, K., HERRMAN, R., MATTERN, J. & VOLM, M. (1985). Sequential methotrexate and 5-fluorouracil in human tumour xenografts. *Med. Oncol. Tumour Pharmacother.*, **2**, 27.